# Correction: Structural basis for the prion-like MAVS filaments in antiviral innate immunity

**DOI:** 10.7554/eLife.07546

**Published:** 2015-08-28

**Authors:** Hui Xu, Xiaojing He, Hui Zheng, Lily J Huang, Fajian Hou, Zhiheng Yu, Michael Jason de la Cruz, Brian Borkowski, Xuewu Zhang, Zhijian J Chen, Qiu-Xing Jiang

**Affiliations:** 1 Department of Cell Biology, University of Texas Southwestern Medical Center, Dallas, United States; 2 Department of Molecular Biology, University of Texas Southwestern Medical Center, Dallas, United States; 3 Department of Pharmacology, University of Texas Southwestern Medical Center, Dallas, United States; 4 CryoEM Shared Resources, Janelia Farm Research Campus, Howard Hughes Medical Institute, Ashburn, United States; 5 Howard Hughes Medical Institute, University of Texas Southwestern Medical Center, Dallas, United States

**Keywords:** MAVS, innate immunity, prion-like filaments, cryoEM reconstruction, human, viruses

Xu H, He X, Zheng H, Huang LJ, Hou F, Yu Z, de la Cruz MJ, Borkowski B, Zhang X, Chen ZJ, Jiang Q-X. 2014. Structural basis for the prion-like MAVS filaments in antiviral innate immunity. *eLife* **3**:e01489. doi: 10.7554/eLife.01489

Published 25 February 2014

Reviewing editor: Wesley I Sundquist, University of Utah School of Medicine, United States

## Introduction

Mitochondrial antiviral signaling (MAVS) protein forms prion-like aggregates mediated by the N-terminal caspase activation and recruitment domain (CARD) and activates antiviral signaling cascades. Purified MAVS CARD from culture cells self-assembles into filaments. Previously, we reported a low-resolution cryoEM structure of MAVS CARD filament, which exhibits a C3 symmetry with a rotation of −53.6° and an axial rise of 16.8 Å for every unit in the filament (Xu et al., 2014). Recently, a cryoEM reconstruction of MAVS CARD filaments at 3.6 Å resolution was reported with a C1 helical symmetry of a rotation of −101.1° and an axial rise of 5.1 Å per subunit (Wu et al., 2014). The differences in these two models were carefully analyzed recently (Egelman, 2014), which suggested that the helical ambiguity in helical reconstruction was not fully resolved in our previous analysis (Xu et al., 2014). We recently collected a new dataset at higher resolutions. Using a newly developed method for analysis of helical filaments (Clemens et al., 2015), we obtained a 4.2 Å resolution reconstruction of MAVS CARD filaments purified from mammalian cells under native conditions. The new model shows that the MAVS CARD filament exhibits a C1 helical symmetry in agreement with Wu et al. (2014).

## Results and discussion

CryoEM images of Flag-tagged MAVS CARD (residues 1–100) protein, which was expressed in HEK293T cells and purified as described previously (Xu et al., 2014), were collected using automated data acquisition in a Titan Krios with a Falcon II direct electron detector (Figure 1A; see ‘Materials and methods’ section for detail). Fourier transforms of motion-corrected raw images showed Thon rings up to ∼3 Å (Figure 1B–C). After CTF fitting, the selected raw images were phase-flipped, and individual filaments were selected interactively using EMAN2 Helixboxer (Ludtke et al., 1999). Selected filaments were segmented using individual boxes that had 90% overlap between neighboring ones. Segmented filament images were high-pass filtered at 120 Å to suppress the low-resolution variations in optical density across individual images as well as the contribution of low-resolution noise to particle alignment. The filament datasets were processed using a customized version of Relion 1.2 (Scheres, 2012; Clemens et al., 2015). This ‘helical’ Relion implemented the Iterative Helical Real Space Reconstruction (IHRSR) method (Egelman, 2010) within the framework of Relion 1.2. For 3D classification and auto-refinement, the helical symmetry was refined by the re-implemented IHRSR module *hsearch* and applied in real space to the 3D volume by the re-implemented IHRSR module *himpose*. The helically symmetrized volume was masked and used as the reference volume in the next iteration (Clemens et al., 2015).

**Figure 1.**
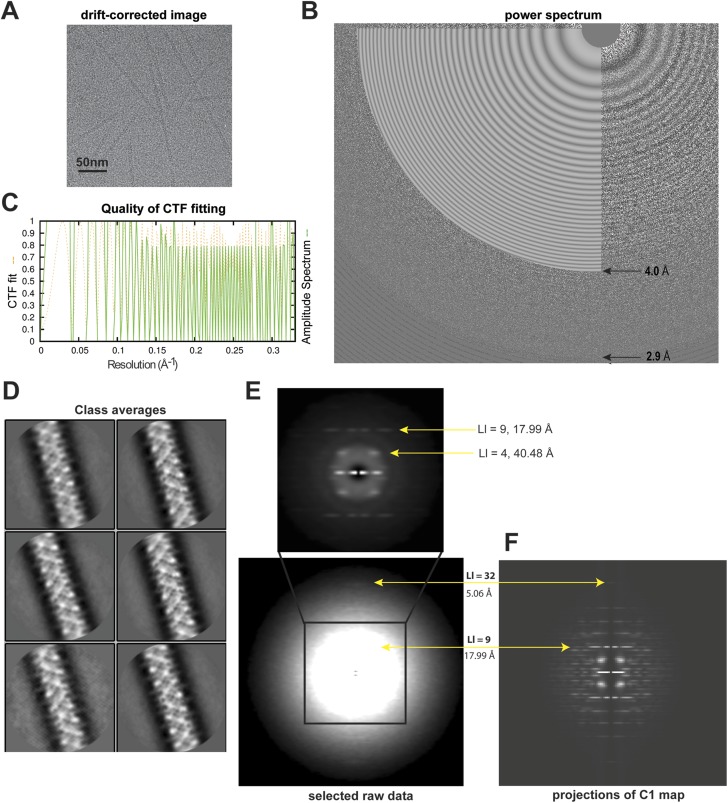
Analyses of high-resolution cryoEM images of MAVS CARD filaments. (**A**) A representative EM image after motion correction. (**B**) The power spectrum of a motion-corrected image showing Thon rings up to ∼3 Å (bottom arrow), with the CTF fittings (white concentric rings) exhibited as white flat annuli over the power spectrum. The CTF fitting was done with CTFFIND4. (**C**) The quality of the CTF fitting was evaluated by comparing the rotationally averaged amplitudes from the Thon rings (green trace) with the averaged amplitudes of the fitted CTF function (dashed yellow trace). The Thon rings are visible to ∼3 Å, and the fitting is good to at least ∼4 Å (top arrow in B). (**D**) Representative class averages of the new dataset showing some fine structural features. The classification was done with Relion 2D classification. (**E**) Summed power spectra of filaments with less than 5° of out-of-plane tilt in the last step of refinement. Layer lines 9 and 32 (meridional) are labeled (yellow arrows). The contrast is enhanced to show layer line 32. The middle portion of the power spectrum is shown as a zoomed-in view to the top with layer lines 4 and 9 labeled. (**F**) Summed power of projections from the C1 map along 360 different directions normal to its helical axis. **DOI:** http://dx.doi.org/10.7554/eLife.07546.001

Particle images were first subjected to reference-free 2D classification and images in good classes showing fine structural details were selected for 3D classification and refinement (Figure 1D). 15,600 out of 27,915 boxed filament segments were used to calculate the final refined map. The summed power spectrum of those filaments showing less than 5° of out-of-plane tilting in the final refinement step (Figure 1E) was calculated and compared with the summed power of the C1 map projections from 360 different directions normal to its helical axis (Figure 1F), and the layerline patterns were similar.

Due to possible symmetry ambiguity, we tested two sets of starting parameters for helical reconstruction (see ‘Materials and methods’ section for detail). Reference models of C1 and C3 symmetries were calculated from our SPIDER-based analysis using sorted fractions of filament particles from the four datasets in our previous paper (Xu et al., 2014). They were filtered to 60 Å and then refined against the new dataset in helical Relion. We found that the new dataset converged better to the C1 symmetry. To reduce model bias, we also calculated a reconstruction by imposing the C1 symmetry for the first five runs of refinement to a C3 model obtained from our original analysis (Xu et al., 2014). The initial C1 symmetry parameters of a twist angle (ΔΦ) = −101.25° and an axial rise (Δz) = 4.75 Å (from our old datasets and adjusted to 4.95 Å due to the change of the imaging system) were derived from the corresponding selection rule defined by indexing the power spectrum. After the fifth run, the refinement was continued to optimize the helical parameters and converged to (ΔΦ = −101.21° and Δz = 5.06 Å) (Figure 2A), close to the C1 symmetry reported by Wu et al. (2014). The final map has an overall resolution of 4.18 Å, estimated from the gold-standard Fourier Shell Correlation (FSC) calculated between two maps that were refined independently against the top and bottom halves of the dataset (Figure 2B; see ‘Materials and methods’ section). To examine the resolution with an independent method, we generated a B-factor weighted map based on the PDB model from Wu et al. (2014) (PDB ID: 3J6J), and used it to calculate FSC against the experimental map. A 0.5 threshold found an estimated resolution of 4.25 Å (Figure 2C), close to the one estimated by using two halves of experimental data (Figure 2B). The new map showed clear grooves in α-helices and multiple bulky side chains (Figure 2D, Video 1).

**Figure 2.**
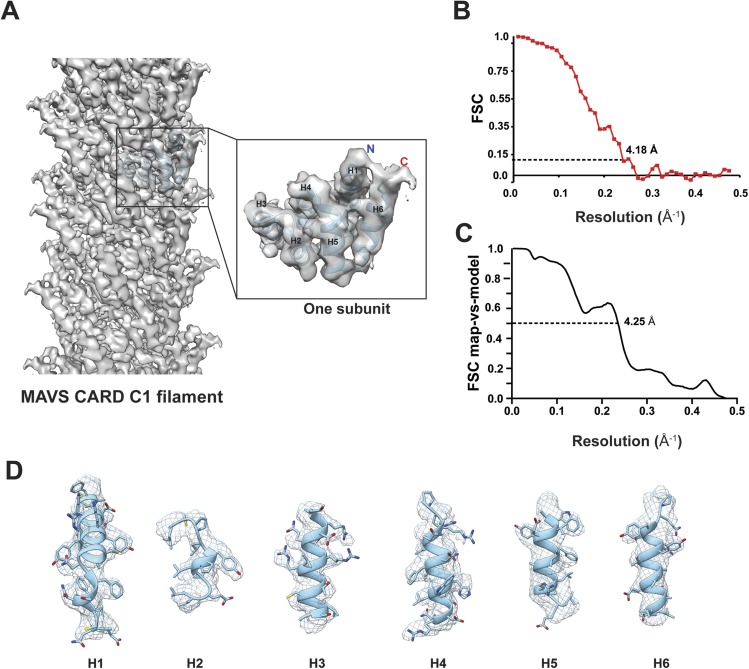
Molecular modeling based on the near-atomic resolution cryoEM map. (**A**) A side view of the cryoEM map with an atomic model of human MAVS CARD docked as a rigid body (blue: atomic model of residues 1–97, PDB: 3J6J). The N- and C- termini face the periphery of the filament. (**B**) Gold-standard Fourier Shell Correlation (FSC) curve between maps calculated from the top and bottom two halves of the selected data. The dashed line points to the estimated resolution of 4.18 Å at FSC = 0.143. (**C**) FSC between the segmented cryoEM map of one CARD and the map calculated from the atomic model (PDB: 3J6J, residues 1–97). The 0.5 thresholding yielded an estimated resolution of 4.25 Å. (**D**) Zoomed-in views of the six α-helices with side chains shown as stick models and superimposed with the EM density (grey mesh). **DOI:** http://dx.doi.org/10.7554/eLife.07546.002

**Video 1.** CryoEM structure of the MAVS CARD filament. **DOI:** http://dx.doi.org/10.7554/eLife.07546.003

The crystal structure of MAVS CARD (Potter et al., 2008) was docked into the density by rigid-body fitting. While the six α-helices of the crystal structure of MAVS CARD monomer (PDB: 2VGQ, residues 3–93) fitted into the EM map well, multiple bulky side chains and those residues in the loop between H4 and H5 needed adjustment. The atomic model reported previously (PDB: 3J6J, residues 1–97, Wu et al., 2014) fit into the density well except for some unoccupied densities next to the N- and C-termini of the docked CARD model (Figure 2A,D). These extra densities may be due to the residues from the N-terminal Flag tag and the additional three residues at the C-terminus of the protein used for preparing the cryoEM specimens; these extra residues are not present in the protein sequence shown in the atomic models.

From the high resolution C1 map, each MAVS CARD monomer interacts with six nearby monomers using three types of intermolecular interfaces (I, II and III) (Figure 3A; Wu et al., 2014).

**Figure 3.**
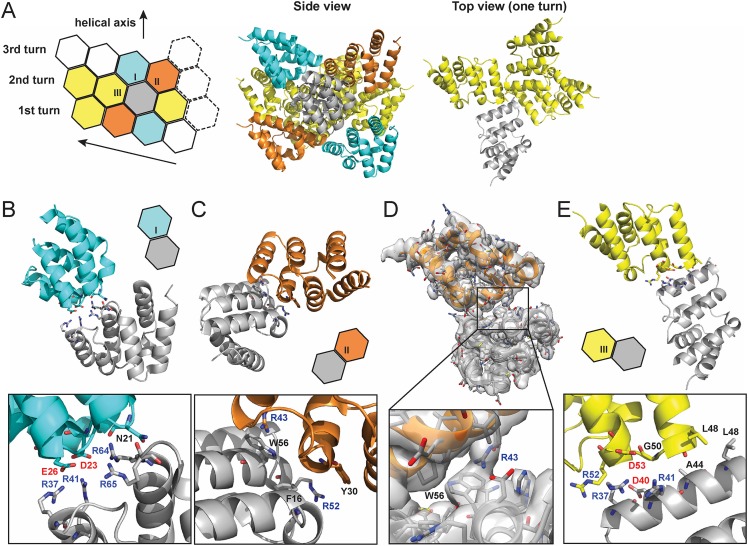
Molecular interfaces of the pseudoatomic model based on the C1 map. (**A**) A model of the MAVS CARD filament based on the cryoEM map, showing three helical turns. Each MAVS CARD interacts with six nearest neighbors: two neighbors along the helical trajectory (grey and yellow), and four neighbors between helical turns (grey and cyan, grey and orange). (**B**, **C**) Details of interactions at type I and II interfaces. Residues at the interfaces are shown as stick models. (**D**) The segmented cryoEM map with two MAVS CARD molecules and a magnified view of type II interface with clear EM density for W56 and R43. (**E**) Important residues at type III interface shown as stick models. **DOI:** http://dx.doi.org/10.7554/eLife.07546.004

At interface I, the positive and negative charges are distributed at two opposite ends of each CARD subunit, indicating that this interface is mainly mediated by electrostatic interactions (Figure 3B). The positively charged residues R37 and R41 in H3, R64 and R65 in the loop between H4 and H5 from one CARD molecule (grey in Figure 3B) are in close proximity to the negatively charged residues D23 in the loop between H1b and H2 and E26 in H2 of another CARD molecule (cyan in Figure 3B). Compared with the wild-type MAVS, which potently induced IFNβ in a dose-dependent manner, D23A showed significantly decreased activity and mutations of other charged residues (E26, R37, R41, R64 and R65) at the interface to alanine or residues with reversed charge almost completely abolished MAVS activity (Xu et al., 2014).

In the crystal structure of MAVS CARD, the aromatic chain of W56 adopts two alternative conformations, with a major conformer (60% occupancy) exposed to solvent and stacked against the ring structure of residue F16 in immediate vicinity (Potter et al., 2008). In the cryoEM map, the density of one conformer is resolved (Figures 2D, 3D) and there appears density corresponding to the side chain of R43 from an adjacent CARD (Figure 3D), suggesting a possible cation–pi interaction between these two residues, as reported previously (Wu et al., 2014). Alanine substitution of R43 almost completely abolished MAVS activity (Xu et al., 2014). Besides W56, the ring structures of residues F16 and Y30 also appear to be important for filament assembly. Single alanine substitution of F16, W56 or Y30 almost completely abolished MAVS activity, while substitutions with different ring-containing residues to these positions, such as F16H, W56F, W56Y, Y30F and Y30H, were able to largely retain MAVS activity (Xu et al., 2014). Another residue involved in stabilizing the interface is R52, whose replacement by alanine significantly decreased its activity (Xu et al., 2014).

Interface III mediates the interactions between adjacent monomers along the helical trajectory. Close to the central pore of the helical filament, residue L48 from each MAVS CARD monomer contributes to the formation of a hydrophobic core with G50 and A44 in close proximity (Figure 3A, top view and Figure 3E). Substitution of L48 with charged residues (D or K), but not alanine, completely abolished MAVS activity in HEK293T cells (Xu et al., 2014). It is interesting to note that L48 is not conserved, and is R or Q in MAVS from multiple other species. The filaments might need to rearrange in order to accommodate such substitutions. The small side chain or hydrophobicity of A44 may be important for the packing at this hydrophobic interface because its mutation to T impaired MAVS CARD filament formation in vitro (Xu et al., 2014). Charged residues R37, R41, D40 and D53 are located at the outer rim of the filament forming electrostatic interactions together with charged residues at type I interface. Single alanine substitution of these residues almost completely abolished MAVS activity (Xu et al., 2014).

Overall, the loss-of-function mutation data are consistent with both the C1 map reported here and the C3 map obtained previously (Xu et al., 2014). However, although key residues involved in the interactions are almost identical in the two models, the detailed interactions at the interfaces are different according to the two maps, indicating that mutagenesis analyses alone cannot distinguish one model from the other.

### Conclusions

MAVS CARD filaments from our new dataset converged to almost the same C1 symmetry as reported by Wu et al. (2014), not the C3 symmetry as we reported previously (Xu et al., 2014). The heterogeneity of the samples and the limited resolution of images were likely among the factors that led to the exclusion of the C1 model in the original analysis. We wish to correct this mistake and apologize for any confusion it might have caused. We stand by all other data in the paper, including in vitro mapping of the oligomerization interface, cell-based mutational analyses, super-resolution SIM imaging of MAVS aggregates in virus-infected cells, and the crystal structures. These experiments for the first time identified most of the key residues involved in MAVS polymerization, leading to an extensive mapping of the molecular surfaces for forming the MAVS filament.

## Materials and methods

### Sample preparation

Flag-MAVS CARD (1–100) expressed in HEK293T cells was purified in a buffer containing 50 mM NaCl. Freshly purified protein was processed for EM experiments to minimize bending and overlapping of filaments on cryoEM grids. To prepare cryo-EM specimens, 2.5 μl freshly purified MAVS CARD (0.1 mg/ml) filaments in suspension were applied to a glow-discharged Quantifoil R2/2 holey carbon grid (Quantifoil Micro Tools GmbH, Jena, Germany) coated with a thin carbon film and plunge-frozen inside a Vitrobot (FEI, Hillsboro, OR).

### Data collection and analysis

Movie data were collected in a FEI Titan Krios microscope equipped with a Falcon II detector at the HHMI Janelia Farm Research Campus. The microscope was equipped with a Cs corrector (Cs = 0.01 mm) and operated at 300 kV. EPU (FEI) was run to control automatic data collection across multiple preselected holes in the holey grids. Images were taken using a defocus range of −2.5 to −4.0 microns at a nominal magnification of 59,000×, which corresponds to a calibrated pixel size of 1.05 Å at the specimen level. Images were taken with a total dose of 35 e^−^/Å^2^ distributed over 16 frames. The movie data were processed using the program dosefgpu_driftcorr (Li et al., 2013). Frames 3–12 of every movie were summed together as the corrected image (Figure 1A). The carbon-support of our filaments made the motion between consecutive frames very small (mostly less than 2 pixels) and we therefore did not do motion correction for individual filaments (polishing) in Relion. We started with 27,916 filaments. After 2D classification, 15,600 filaments were selected. During the 3D classification into two different classes, there was a negligible fraction of the filaments conforming to a different volume with poor alignment statistics. We therefore kept all 15,600 filaments for final map calculation, which correspond to ∼80,000 images of individual CARD units.

In order to calculate a gold-standard FSC from truly independent datasets, we divided the 15,600 filaments into the top and bottom two halves. These two datasets had negligible overlap. Two separate C1 models were generated using the SPIDER-based method from a small sorted fraction (∼13%) of filaments out of our old datasets as described before (Xu et al., 2014), and imposed with diagonally varied symmetry parameters (5.22 Å, −102.2°) and (4.82 Å, −100.2°), respectively. These two models were used as initial models and separately refined against the two half datasets in helical Relion. The two refinements converged to almost the same symmetry parameters, (5.05 Å, −101.25°) and (5.08 Å, −101.20°), respectively. The two volumes were post-processed in EMAN2 and aligned in CHIMERA. The map calculated from the top half of the dataset was rotated −2.4° and shifted by −1.05 pixels before being compared with the one from the bottom half to calculate a gold-standard FSC using e2proc3d.py in EMAN2. The calculations in the final refinement steps were done in the *largemem* nodes of the STAMPEDE supercomputer at the Texas Advanced Computer Center (TACC) in Austin, Texas. Molecular modeling was done in CHIMERA and Coot (Emsley et al., 2010).

## Funding

**Table:** 

Funder	Grant reference	Author
National Institutes of Health	R01GM088745	Hui Xu, Brian Borkowski, Qiu-Xing Jiang
National Institutes of Health	R01GM093271	Hui Xu, Brian Borkowski, Qiu-Xing Jiang
National Institutes of Health	R01GM088197	Xiaojing He, Xuewu Zhang
National Institutes of Health	1S10RR027972	Qiu-Xing Jiang
American Heart Association	12IRG9400019	Qiu-Xing Jiang
Welch	I-1684, I-1389, and I-1702	Hui Zheng, Qiu-Xing Jiang, Hui Xu, Zhijian J Chen, Xuewu Zhang
Cancer Prevention Research Institute of Texas	RP120474	Hui Xu, Qiu-Xing Jiang
National Institutes of Health	R01AI093967	Zhijian J Chen
Howard Hughes Medical Institute		Zhiheng Yu, Michael Jason de la Cruz, Zhijian J Chen
National Institutes of Health	C06RR30414	Hui Xu, Hui Zheng, Lily J Huang, Brian Borkowski, Qiu-Xing Jiang

## References

[bib1] Clemens DL, Ge P, Lee BY, Horwitz MA, Zhou ZH (2015). Atomic structure of T6SS reveals interlaced array essential to function. Cell.

[bib2] Egelman EH (2010). Reconstruction of helical filaments and tubes. Methods in Enzymology.

[bib3] Egelman EH (2014). Ambiguities in helical reconstruction. eLife.

[bib4] Emsley P, Lohkamp B, Scott WG, Cowtan K (2010). Features and development of Coot. Acta Crystallographica. Section D, Biological Crystallography.

[bib5] Li X, Mooney P, Zheng S, Booth C, Braunfeld MB, Gubbens S, Agard DA, Cheng Y (2013). Electron counting and beam-induced motion correction enable near-atomic-resolution single-particle cryo-EM. Nature Methods.

[bib6] Ludtke SJ, Baldwin PR, Chiu W (1999). EMAN: semiautomated software for high- resolution single-particle reconstructions. Journal of Structural Biology.

[bib7] Potter JA, Randall RE, Taylor GL (2008). Crystal structure of human IPS-1/MAVS/VISA/Cardif caspase activation recruitment domain. BMC Structural Biology.

[bib8] Scheres SH (2012). RELION: implementation of a Bayesian approach to cryo-EM structure determination. Journal of Structural Biology.

[bib9] Wu B, Peisley A, Tetrault D, Li Z, Egelman EH, Magor KE, Walz T, Penczek PA, Hur S (2014). Molecular imprinting as a signal-activation mechanism of the viral RNA sensor RIG-I. Molecular Cell.

[bib10] Xu H, He X, Zheng H, Huang LJ, Hou F, Yu Z, de la Cruz MJ, Borkowski B, Zhang X, Chen ZJ, Jiang QX (2014). Structural basis for the prion-like MAVS filaments in antiviral innate immunity. eLife.

