# Supplementary material for: Structural basis for the prion-like MAVS filaments in antiviral innate immunity
Source: eLife. 2014 Feb 25;3:e01489. doi: 10.7554/eLife.01489 (PMC3932521; doi:10.7554/eLife.01489)
Supplement: Supplementary file 1. — DOI: http://dx.doi.org/10.7554/eLife.01489.019 [file elife01489s001.docx]

**Supplemental Table 1.** **Data collection and refinement statistics**

| **Data collection** |  |  |
| --- | --- | --- |
| Crystal | Horse CARD E26R | Horse CARD R64C |
| Space group | P2_1_2_1_2 | P2_1_2_1_2 |
| Cell dimensions |  |  |
| *a*, *b*, *c* (Å) | 50.61, 51.94, 35.75 | 50.82, 52.03, 35.58 |
|  *α* *β* *γ* (°) | 90, 90, 90 | 90, 90, 90 |
| Resolution (Å) | 50.0-1.95(1.98-1.95)* | 50.0-2.35(2.39-2.35)* |
| *R*_sym_ | 9.3(20.6) | 5.9(12.8) |
| *I*/σ*I* | 21.3(6.0) | 42.8(13.5) |
| Completeness (%) | 97.8 (87.4) | 98.8(84.6) |
| Redundancy | 5.9(4.2) | 6.5(4.8) |
|  |  |  |
| **Refinement** |  |  |
| Resolution (Å) | 1.95 | 2.35 |
| No. reflections | 7206 | 4196 |
| Completeness(%) | 98 | 99 |
| *R*_work_/*R*_free_ (%) | 16.3/20.9 | 16.3/22.7 |
| No. atoms | 944 | 839 |
| Protein | 788 | 765 |
| Water | 156 | 74 |
| B-factors |  |  |
| Protein | 9.9 | 20.3 |
| Water | 23.8 | 29.4 |
| R.m.s deviations |  |  |
| Bond lengths (Å) | 0.0074 | 0.0069 |
| Bond angles (°) | 0.94 | 0.96 |
| Ramanchandran plot |  |  |
| Favored (%) | 98.94 | 97.83 |
| Allowed (%) | 1.06 | 2.17 |
| Disallowed (%) | 0 | 0 |

*Highest resolution shell is shown in parenthesis.
